# miR-142 downregulation alleviates rat PTSD-like behaviors, reduces the level of inflammatory cytokine expression and apoptosis in hippocampus, and upregulates the expression of fragile X mental retardation protein

**DOI:** 10.1186/s12974-020-02064-0

**Published:** 2021-01-06

**Authors:** Peng-Yin Nie, Lei Tong, Ming-Da Li, Chang-Hai Fu, Jun-Bo Peng, Li-Li Ji

**Affiliations:** 1 Department of Anatomy, College of Basic Medical Sciences, China Medical University, Shenyang, China; 2 Department of 1st Clinical Medicine, China Medical University, Shenyang, China

**Keywords:** Post-traumatic stress disorder, miR-142, NF-κB, FMRP, Inflammation

## Abstract

**Background:**

FMRP is a selective mRNA-binding protein that regulates protein synthesis at synapses, and its loss may lead to the impairment of trace fear memory. Previously, we found that FMRP levels in the hippocampus of rats with post-traumatic stress disorder (PTSD) were decreased. However, the mechanism underlying these changes remains unclear.

**Methods:**

Forty-eight male Sprague-Dawley rats were randomly divided into four groups. The experimental groups were treated with the single-prolonged stress (SPS) procedure and injected with a lentivirus-mediated inhibitor of miR-142-5p. Behavior test as well as morphology and molecular biology experiments were performed to detect the effect of miR-142 downregulation on PTSD, which was further verified by in vitro experiments.

**Results:**

We found that silence of miRNA-142 (miR-142), an upstream regulator of FMRP, could alleviate PTSD-like behaviors of rats exposed to the SPS paradigm. MiR-142 silence not only decreased the levels of proinflammatory mediators, such as interleukin-1β, interleukin-6, and tumor necrosis factor-α, but also increased the expressive levels of synaptic proteins including PSD95 and synapsin I in the hippocampus, which was one of the key brain regions associated with PTSD. We further detected that miR-142 silence also downregulated the transportation of nuclear factor kappa-B (NF-κB) into the nuclei of neurons and might further affect the morphology of neurons.

**Conclusions:**

The results revealed miR-142 downregulation could alleviate PTSD-like behaviors through attenuating neuroinflammation in the hippocampus of SPS rats by binding to FMRP.

## Introduction

Post-traumatic stress disorder (PTSD) is one of the psychiatric disorders related to pathological fear and anxiety [1, 2]. It often presents a series of mental symptoms including re-experiencing, avoidance/numbing, hyperarousal, negative cognition, and mood following exposure to a life-threatening event that can result in a psychological trauma, such as combat, interpersonal violence, and natural disasters [3]. It has been reported that potential alterations in neuroendocrine, psychophysiological, and neurobiological systems were also related to the etiology and maintenance of PTSD [4]. Some studies have found that patients with PTSD showed elevated blood levels of proinflammatory mediators, including interleukin-1β (IL-1β), interleukin-6 (IL-6), tumor necrosis factor-α (TNF-α), and C-reactive protein (CRP), accompanied with immune dysregulation and inflammation [5–8]. Neuroinflammation is characterized by the activation of glial cells (mainly astrocytes and microglia), which release various soluble factors, including cytokines, reactive oxygen species (ROS) and reactive nitrogen species (RNS), and lipid metabolites. Most of these glia-derived factors are proinflammatory and neurotoxic and are particularly deleterious to neurons [9, 10]. Microglia are rapidly activated and acquire the ability to release various inflammatory molecules that influence neuronal survival, as well as synaptic function and plasticity in the event of pathological insults [11].

Fragile X mental retardation protein (FMRP) is a selective mRNA-binding protein that participates in the regulation of gene expression by binding to mRNA and adjusting their translational efficiency and trafficking [12, 13]. It has been reported that FMRP could regulate protein synthesis in synapses, and its loss could cause the impairment of trace fear memory [14, 15]. In addition, it has been shown that FMRP was present in dendritic spines and could mediate mRNA translation in dendrites [16–18]. The effect of FMRP loss on spine phenotypes in the hippocampus was found to be diverse and, for some studies, showed an impairment in spine maturation, while others failed to find this [19–21]. Our previous study found that the level of FMRP in the hippocampus of rats exposed to the single-prolonged stress (SPS) was downregulated, suggesting that FMRP might be involved in the pathophysiological process of PTSD, but the specific mechanism has not been studied.

MicroRNAs (miRNAs), which play crucial roles in post-transcriptional gene regulation of several physiological and pathological processes, are small noncoding RNA molecules that are widely expressed and dysregulated in the early stage of diseases by altering the expression of target genes or proteins [22, 23]. Changes in the expression of MiRNAs lead to neuronal dysfunction in diseases of the central nervous system (CNS) and have emerged as important regulators of neuronal function [22, 24]. Among these miRNAs, our previous studies showed that miR-142 in the amygdala of PTSD rats was upregulated, and inhibition of miR-142 could reduce anxiety-like behavior and memory deficits by targeting Npas4 [25]. However, whether other target genes of miR-142 play a role in regulating PTSD-like behaviors in downstream mechanisms still needs further study. TargetScan predicts biological targets of miRNAs and suggests that FMRP is a putative target of miR-142 (http://www.targetscan.org). As both miR-142 and FMRP have associations with PTSD, we were keen to know whether miR-142 might contribute to the pathological process of PTSD-like pathology by regulating FMRP and downstream molecules.

In the present study, we found that miR-142 and proinflammatory cytokines, including TNF-α, IL-1β, and IL-6, were increased in the hippocampus of SPS rats compared to normal rats. MiR-142 downregulation could alleviate PTSD-like behaviors, such as anxiety, depression, and memory disorders, in rats exposed to SPS procedure by combing with FMRP, which might regulate the level of pre-inflammatory chemokines by affecting nuclear factor kappa-B (NF-κB) signaling and then affect synaptic function. Furthermore, we clarified that downregulation of miR-142 could reduce the inflammatory response of neuron-like cells cultured in conditioned medium (CM) of lipopolysaccharide (LPS)-treated microglia through the NF-κB signaling pathway. This study provides the mechanism of action of miR-142 in neurons and microglia under inflammatory conditions and may become a potential new therapeutic target for the treatment of PTSD.

## Methods

### Animals

Forty-eight male Sprague-Dawley rats, obtained from the Experimental Animal Center of China Medical University, were of the same age (3–4 months) and weighed about 200 g. All animals were fed in groups of four per cage under standard laboratory conditions (constant temperature ranged from 22 to 26 °C, air humidity ranged from 40 to 60%) with water and food freely available under a 12-h light/dark cycle. All experimental procedures on animals, which carried out following an adaptation period for at least 1 week, was approved by the Institutional Animal Care and Use Committee (China Medical University, China), which was in accordance with the National Institutes of Health Guide for the care and use of laboratory animals.

### Experimental groups and single-prolonged stress (SPS) procedure

**Fig. 1:**
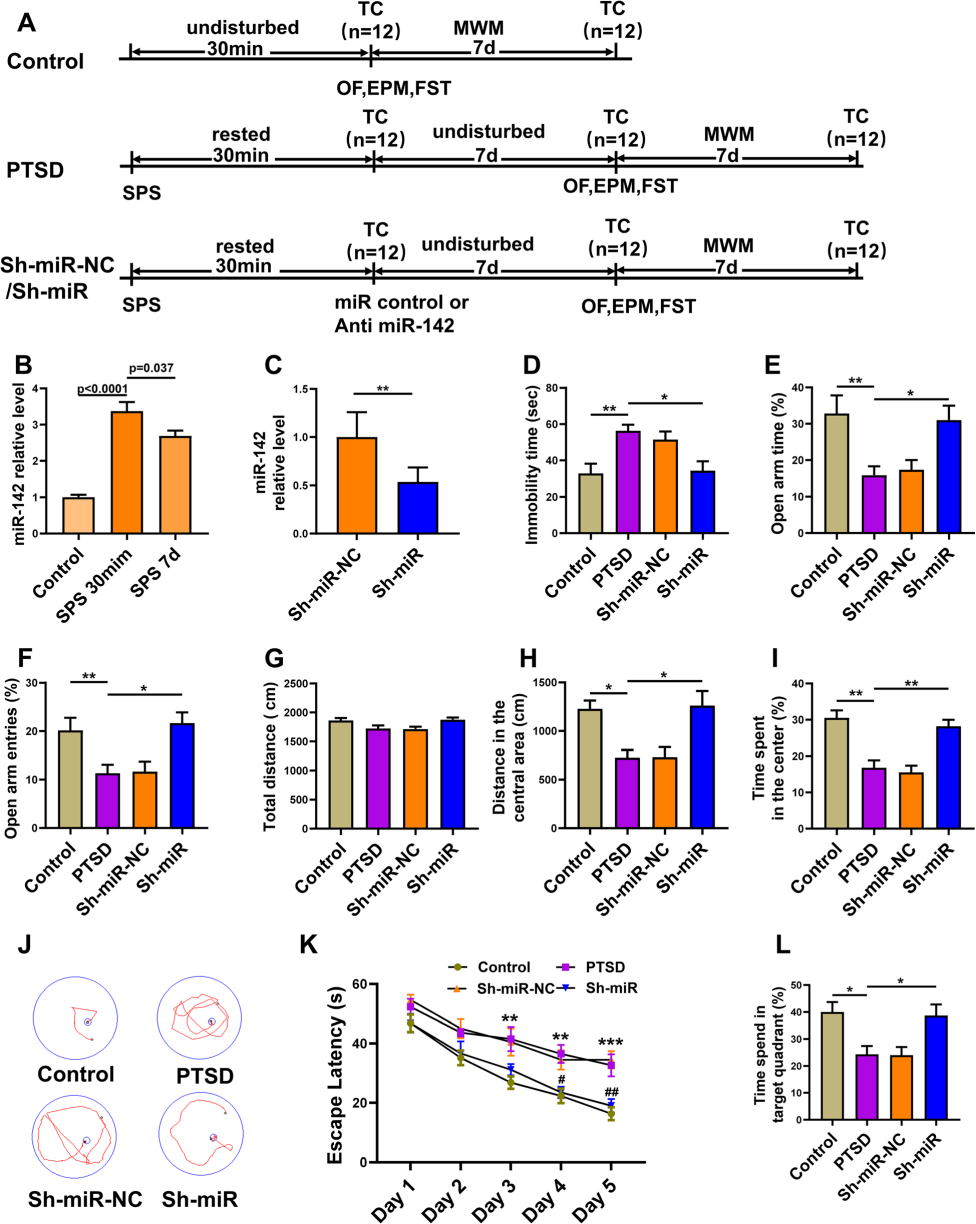
The antisense miR-142-5p alleviates the PTSD-like behavior in rats exposed to the SPS procedure. **a** The procedure and timeline of PTSD experiments. **b** RT-qPCR detection of relative expression of miR-142 in rat hippocampus at different time points. **c** The relative expression miR-142 in rat hippocampus tested by RT-qPCR two weeks after LV-anti-miR-142 administration. **d** The immobility time in FST. **e** Open arm time and **f** open arm entries in the EPM test. **g** Representative tracts on day 5 during navigation trials, **h** the mean escape latency during the five training days, and **i** the percentage of time spent in the target quadrant in the MWM test. **j** Total distance, **k** distance in the central area, and **l** the percentage of time spent in the center of rats in the OF test. *n* = 6 rats per genotype. Bar graphs represent the mean ± SEM of independent experimental triplicates. One-way or two-way ANOVA followed by Bonferroni’s post hoc test; *P < 0.05, **P < 0.01

The animals were randomly divided into four groups as follows: control group, all animals were normal rats (*n* = 12); PTSD group, in which rats received SPS treatment (*n* = 12); Sh-miR-NC group, in which SPS rats were given scramble control lentivirus (LV-NC, *n* = 12); Sh-miR group, where SPS rats were treated with miR-142 reverse complementary sequence encoding lentivirus (*n* = 12) (Fig. 1a). The rats then performed the following experimental procedures, including behavioral tests, immunofluorescence staining, and western blotting. Six of the rats were used for immunofluorescence staining, and the other six were used for western blotting and enzyme-linked immunosorbent assay.

The SPS procedure was performed as previously described by Liberzon et al. [26]. Briefly, rats were restrained in a disposable restraint holder (58 mm in diameter and 150 mm in length) for 2 h, and then the animals were forced into a transparent plastic container (600 mm × 400 mm × 500 mm with 24 °C water) swimming for 20 min. After that, the rats were allowed to recover for 15 min and then exposed to ether until they lost consciousness. The animals were kept in their cages for 7 days without interference, and then they were subjected to behavioral tests.

### Injection of lentivirus-mediated inhibitors of miR-142-5p

After anesthetized with pentobarbital sodium, the rats were fixed in a stereotaxic apparatus. Afterwards, the miR-142-5p lentivirus-encodedreverse complementary sequence (LV-anti-miR-142; Wanlei Biotechnology Co., Ltd.; Shenyang, China) or scramble control (LV-NC; Wanlei Biotechnology) were infused into the right lateral ventricle of rats (1.2 mm posterior to the bregma, 1.8–2.0 mm right to sagittal suture, and 4.0 mm below the external surface) at a dose of 10 μl [27, 28]. The lentivirus titers used for the experiments were 1 × 10^8^ transducing U/ml.

### Behavioral paradigms

The purpose of the behavior test was to detect the effect of downregulation of miR-142 on PTSD. Six rats per group were acclimated in the laboratory for 2 h before starting the test. Experimental methods included forced swimming test (FST), elevated plus maze (EPM) test, open field test (OFT), and Morris water maze (MWM), allowing animals to rest for at least half an hour between each stage.

### Forced swim test (FST)

FST was used to evaluate depression-like behavior in rats. The rats were placed in a plastic container (20 cm in diameter and 40 cm in height) filled with water (23–25 °C, 30 cm water depth) and swim for 6 min. The rats were relatively active within the first 2 min. They were assessed as immobile when they stopped struggling and remained passively floating to keep their heads above the water and did not attempt to escape within the last 4 min.

### Elevated plus maze (EPM) test

The EPM test was used to assess anxiety-like behavior and was performed after OFT. In short, the rats were placed on the central platform facing an open arm and allowed to explore for 5 min. Anxiety behavior of animals was quantified by the percentage of time spent in the open arms and the percentage of entries into the open arms with respect to total (open + closed) arms.

### Open field test (OFT)

OFT was used to evaluate anxiety-like behavior in rats. Each animal was placed in the corner of the arena to freely explore the device (720 mm × 720 mm × 40 mm) for 5 min.

The spontaneous locomotor activity and duration in the center area (the four squares in the center of apparatus) and the total immobility time of rats were recorded.

### Morris water maze (MWM)

MWM was used to evaluate the spatial learning, memory, and cognitive ability of rats. In short, the MWM test was carried out in a circular water tank (120 cm diameter, water temperature 24 ± 1 °C), and the water surface height exceeded 2 cm above the platform. During the first 5 consecutive days, the rats underwent navigation test three times a day. On the first day of training, the rats could see where the submerged platform was, and then they were placed in a random quadrant, with the nose facing the wall. The platform was hidden underwater on other days. The escape latency was measured by the time the animal sought the hidden platform. If the animals could not escape within 60 s, they would be directed to the platform and stay for 10 s. On the last day, the platform was removed, and the probe test was used to assess the spatial memory of the animal.

### Cell culture and transfection

#### Hippocampal primary neuronal cells cultures

The hippocampus of six newborn rats was put into Ca^2+^/Mg^2+^-free Hank’s balanced salt solution (HBSS) and cut into 1-mm^3^ pieces. The tissue was placed in PBS containing 0.25% trypsin for 15 min at 37 °C, and then digestion was stopped with a medium containing serum. After centrifugation, the cells were resuspended and blown into a single-cell suspension at a density of 1 × 10^6^ cells/ml. Cells were then seeded on poly-l-lysine-coated (1 mg/ml; Sigma-Aldrich, MO, USA) glass coverslips on a 6-well culture plate, and incubated in neural-basal (Gibco, Grand Island, NY, USA) containing 2% B27 supplement (Gibco) at 37 °C in 5% CO_2_ and 95% air with saturated humidity. The culture medium was changed every other day.

#### Primary microglial cells culture and conditioned media collection

The cerebral cortex extracted from six newborn rats was put into Ca^2+^/Mg^2+^-free Hank’s balanced salt solution and minced with a sterile scalpel. The tissue was placed in PBS containing 0.25% trypsin for 15 min at 37 °C, and then digestion was stopped with a medium containing serum. After centrifugation, the cells were resuspended and blown into a single-cell suspension at a density of 1 × 10^6^ cells/ml. After 24 h, the non-adherent cells were removed and the medium was completely replaced. The cells were shaken on a benchtop concentrator for 15 min, and the first half of the cells were incubated with Dulbecco’s modified Eagle medium/Ham’s nutrient mixture F-12 (DMEM/F12, 1:1, Gibco) containing 10% fetal bovine serum (FBS, Gibco), 10% B27 (Gibco), 10% N2 supplement (Gibco), and 1% penicillin-streptomycin (P/S) at 37 °C in 5% CO_2_ and 95% air with saturated humidity. Half of the cells were incubated for 24 h in a medium containing lipopolysaccharide (LPS, Sigma-Aldrich Corp., St. Louis, Missouri, USA) at a concentration of 500 ng/mL [29]. Conditioned media from above cells (CM) and LPS-treated cell conditioned media (L-CM) were collected.

#### Cell induction and transfection

The PC12 cell line derived from rat adrenal medulla was a kind gift provided by Dr. Li Bin, China Medical University, and maintained in culture medium including Dulbecco’s modified Eagle medium (DMEM, Gibco), 10% fetal bovine serum (FBS, Gibco), and 1% penicillin-streptomycin (P/S) at 37 °C in 5% CO_2_ and 95% air with saturated humidity. The culture medium was changed to DMEM without FBS 12 h before drug treatments and then incubated in the DMEM contained 100 ng/mL nerve growth factor (NGF, R&D, Minneapolis, MN, USA) for 24 h to obtain the neuron-like cells. The neuron-like cells were cultured in medium containing microglia conditioned medium (CM) or LPS-induced microglia conditioned medium (L-CM). LV-anti-miR-142 (sh-miR group) and LV- (sh-miR-NC group) were transfected into cells for 72 h. The cells in sh-miR group and sh-miR-NC group were also cultured in L-CM. After continuous culture for 1 week, the total protein or nuclear protein of the cells was extracted for subsequent experiments.

### Immunofluorescence staining

Rats were transcardially perfused with saline and fixed by perfusion of 4% paraformaldehyde (PFA) dissolved in phosphate buffer saline (PBS) under deep anesthesia by intraperitoneal injection of pentobarbital sodium at a dose of 150 mg/kg. The brains were immersed into 4% PFA overnight after extraction, and then the tissue was cut into 7-μm-thick sections after gradient sugar precipitation. The sections were washed in 0.1 M PBS for three times and by 0.2% Triton X-100 for 30 min at room temperature. After blocking with 5% permeabilized bovine serum albumin (BSA), the sections were immersed in the following primary antibodies (all from Abcam) overnight at 4 °C: mouse β-tubulin primary antibody (1: 500), rabbit FMRP primary antibody (1: 300), goat Iba-1 (1: 500) primary antibody, rabbit TNF-α primary antibody (1: 300), rabbit IL-1β primary antibody (1: 300), rabbit IL-10 primary antibody (1: 300), rabbit PSD95 primary antibody (1: 200), and goat synapsin I primary antibody (1: 200). The sections were then incubated in the following secondary antibody (all from Abcam) solutions for 2 h: goat anti-mouse 488-conjugated secondary antibody (1: 600), donkey anti-rabbit 594-conjugated secondary antibody (1: 600), and donkey anti-goat 594-conjugated secondary antibody (1: 600), and then incubated in 100 ng/ml DAPI to stain the nuclei for 5 min. Slides were fixed with 50% glycerol, and images were captured by a confocal laser microscope (FV1000, Olympus) using standard laser lines and filters. The cultured cells were fixed with 4% PFA at room temperature for 40 min. The procedure of immunofluorescence staining was the same as that of tissue.

### Western blotting analysis

The hippocampus of 6 rats in each group was stripped on ice and dissected. After washing three times with PBS, total protein and nuclear protein were extracted with radioimmunoprecipitation assay buffer (RIPA, Beyotime Biotech, Shanghai, China) and nuclear protein extraction kit (Beyotime). The protein concentration was evaluated by the Bicinchoninic acid (BCA) Protein Assay Kit (Beyotime); the same amount of protein (30 μg) from each group was added to the wells and separated by 8% electrophoresis SDS-PAGE gel. The protein was then transferred to a polyvinylidene fluoride (PVDF) membrane (Millipores, Billerica, MA, USA) and blocked with 5% BSA dissolved in the stripping solution at 37 °C for 1 h. The membrane was incubated with primary antibodies that recognize Iba-1, IL-1β, IL-10, IL-6, TNF-α, iNOS, PSD95, Synapsin I, and NF-κBp65 (all diluted to 1:1000 and purchased from Abcam), PARP, FMRP (1:10,000, abcam) and GAPDH (1:10,000, Proteintech Biotech, Wuhan, China) overnight at 4 °C. After washing 3 times with stripping buffer containing 1% Tween 20, the membrane was incubated with the corresponding secondary antibody at 37 °C for 1 h. The protein bands were visualized by enhanced chemiluminescence (ECL; Uscn Life, China) on a ChemiDoc XRS + imaging system (Bio-Rad, USA).

### Real-time quantitative PCR (RT-qPCR)

Total RNA isolation kit (Vazyme Biotech, Nanjing, China) was used to extract total RNA from rat hippocampus, and reverse transcription (RT) was performed using miRNA first strand cDNA synthesis kit (Vazyme Biotech). Amplification reactions were performed using the StepOnePlus system (Applied Biosystems, USA) and corresponding primers obtained from Sangon Biotech (Shanghai, China). The primer sequences of miR-142-5p: Forward, 5′-GGCCCATAAAGTAGAAAGC-3′; Reverse, 5′-TTTGGCACTAGCACATT-3′; U6 was used as a loading control and the primer sequences of U6 was listed as follows: Forward, 5′-CTCGCTTCGGCAGCACATA-3′; Reverse, 5′-AACGATTCACGAATTTGCGT-3′. The 2^−△△Ct^ method was used to analyze fold changes.

### Flow cytometry

The level of apoptosis was assessed by the PE Annexin V Apoptosis Detection Kit (BD Biosciences, Burlington, MA, USA) according to the manufacturer’s instruction. Briefly, the hippocampus of rats in each group was dissected and made into a single-cell suspension. The cells were washed twice with cold PBS and then resuspended by binding buffer at a concentration of 1 × 10^6^ cells/ml. The cell solution (1 × 10^5^ cells) was transferred into a 5-ml culture tube.

Then, a mixture of 5 μl phycoerythrin (PE)-labeled Annexin V and 5 μl 7-aminoactinomycin D (7-AAD) was added to the culture tube. The cells were gently vortexed and incubated for 15 min at room temperature (25 °C) in the dark. After adding 400 μl of binding buffer to each tube, the apoptotic cells were analyzed with a FACSCalibur® flow cytometer. PE-labeled Annexin V positive cells were considered to be apoptotic cells, and dead cells that were positive for nuclear dye 7-AAD were also detected. After the designated treatment, the number of dead, late apoptosis, early apoptosis, and living cells was counted.

### Enzyme-linked immunosorbent assay

ELISA method was used to measure the content of IL-1β, IL-6 (both obtained from CUSABIO, Wuhan, China), and IL-10 (BOOST Biotech, Wuhan, China) in rat hippocampus exposed to the SPS paradigm according to the manufacturer’s instructions of kits. Briefly, the hippocampus was separated and homogenized in PBS containing protease inhibitors. The tissue homogenate was centrifuged at 13,000×*g* for 20 min at 4 °C, and the supernatant was collected and immediately stored at − 80 °C until further processing.

### Luciferase reporter assay

The direct binding ability between miR-142-5p and FMRP was verified by luciferase reporter assay. The wild-type FMRP 3′UTR reporter plasmid (pmiR-FMRP-wt) and mutant FMRP 3′UTR reporter plasmid (pmiR-FMRP-mut) were constructed by GenePharma (Shanghai, China). When the cells seeded in the 24-well plate reached approximately 70% confluence, the pmiR-FMRP-wt or pmiR-FMRP-mut and miR-142 mimics or scrambled RNA were co-transfected into HEK 293 T cells with Lipofectamine 2000 (Invitrogen, USA), according to the instructions of the manufacturer of the dual-luciferase reporter assay kit (Promega, USA).

The relative luciferase activity of firefly luciferase was evaluated by the ratio of the fluorescence intensity of firefly luciferase activity to that of Renilla luciferase activity and compared with the empty control. The experiment was performed in quadruplicate and repeated at least three times.

### Statistical analysis

All data were analyzed with SPSS19.0 statistical software, and the measured data were expressed as mean ± SEM (standard error of mean). Statistical comparisons between groups were performed by independent sample *t* test. One-way or two-way analysis of variance (ANOVA) and Bonferroni’s post hoc test analysis were used to analyze the data between the groups. In all cases, *P* < 0.05 was considered a significant difference.

## Results

### Antisense miR-142-5p alleviated PTSD-like behavior in rats exposed to the SPS procedure

After administering antisense miR-142-5p delivered by LV-anti-miR-142 or LV-Control through the lateral ventricle, different behavioral tests were performed and the rats were sacrificed at different time points from other experimental procedures (Fig. 1a). Thirty minutes or 7 days after SPS surgery, the expression of miR-142 in the hippocampus of the control and PTSD groups was measured by RT-qPCR analysis, because miR-142 was reported to be one of several dysregulated miRNAs after traumatic stress exposure. Exposure is consistent with our previous report that its expression was upregulated 7 or 14 days after the SPS paradigm [25]. Indeed, compared with the control group, the level of miR-142 in the PTSD group was increased significantly (*F*(2, 15) = 51.22, *P* < 0.0001, Fig. 1b), suggesting miR-142 might be involved in the pathogenesis of PTSD. Based on the results of RT-qPCR analysis, we injected miR-142-5p antisense delivered by the lentivirus in the right ventricle to test the role of miR-142 in PTSD-like disorders 30 min after SPS surgery. The transfection efficiency was evaluated by RT-qPCR after administration, as shown in Fig. 1c.

Different behavioral tests were used to evaluate the effect of antisense miR-142-5p on the PTSD-like behavior of rats, which include depressive-like behavior, anxiety-like behavior and memory abnormality resulted from the upregulation of miR-142. In FST, we found that the immobility time of SPS rats was longer than that of the control group. However, the immobility time of the rats in the Sh-miR group was shorter than that of the SPS rats in the PTSD group (*F*(3, 20) = 6.627, *P* = 0.0027, Fig. 1d). Then, we conducted an EPM test to assess whether the downregulation of miR-142 could reduce anxiety-like behavior in PTSD rats. The results showed that compared with the control group, SPS rats spent significantly less time on the open arms and fewer entries into the open arms. However, this trend can be reversed by anti-miR-142 injection (*F*(3, 20) = 7.674, *P* = 0.0013, Fig. 1e; *F*(3, 20) = 6.274, *P* = 0.0035, Fig. 1f). In addition, OFT was also used to evaluate the movement and anxiety-like behavior of animals. The results showed that there was no significant difference in the total distance between the four groups, as shown in Fig. 1g. However, rats in the PTSD group traveled significantly shorter distance and spent less time in the central area. The administration of anti-miR-142 significantly reversed this trend (*F*(3, 20) = 7.507, *P* = 0.0015, Fig. 1h; *F*(3, 20) = 12.62, *P* < 0.0001, Fig. 1i).

Finally, we checked whether miR-142 dysfunction had an effect on spatial learning and memory impairment in SPS rats through the MWM test. The escape latency of the PTSD group was significantly longer than that of the control group, and anti-miR-142 could reverse this trend (Fig. 1k). Using a probe test to assess spatial memory, the results showed that the rats in the PTSD group significantly reduced the time spent in the target quadrant, while the anti-miR-142 administration increased the percentage of time (*F*(3, 20) = 6.173, *P* = 0.0038, Fig. 1l). The probe test was used to evaluate spatial memory. The results showed that the rats in the PTSD group significantly reduced the time spent in the target quadrant, while the administration of anti-miR-142 increased the percentage of time.

These results indicated that rats exposed to the SPS procedure exhibited significant depression-like behavior, anxiety-like behavior, and spatial memory disorder, which could be alleviated by administering anti-miR-142 30 min after the SPS procedure.

### The effect of anti-miR-142 on expression of synaptic proteins

**Fig. 2:**
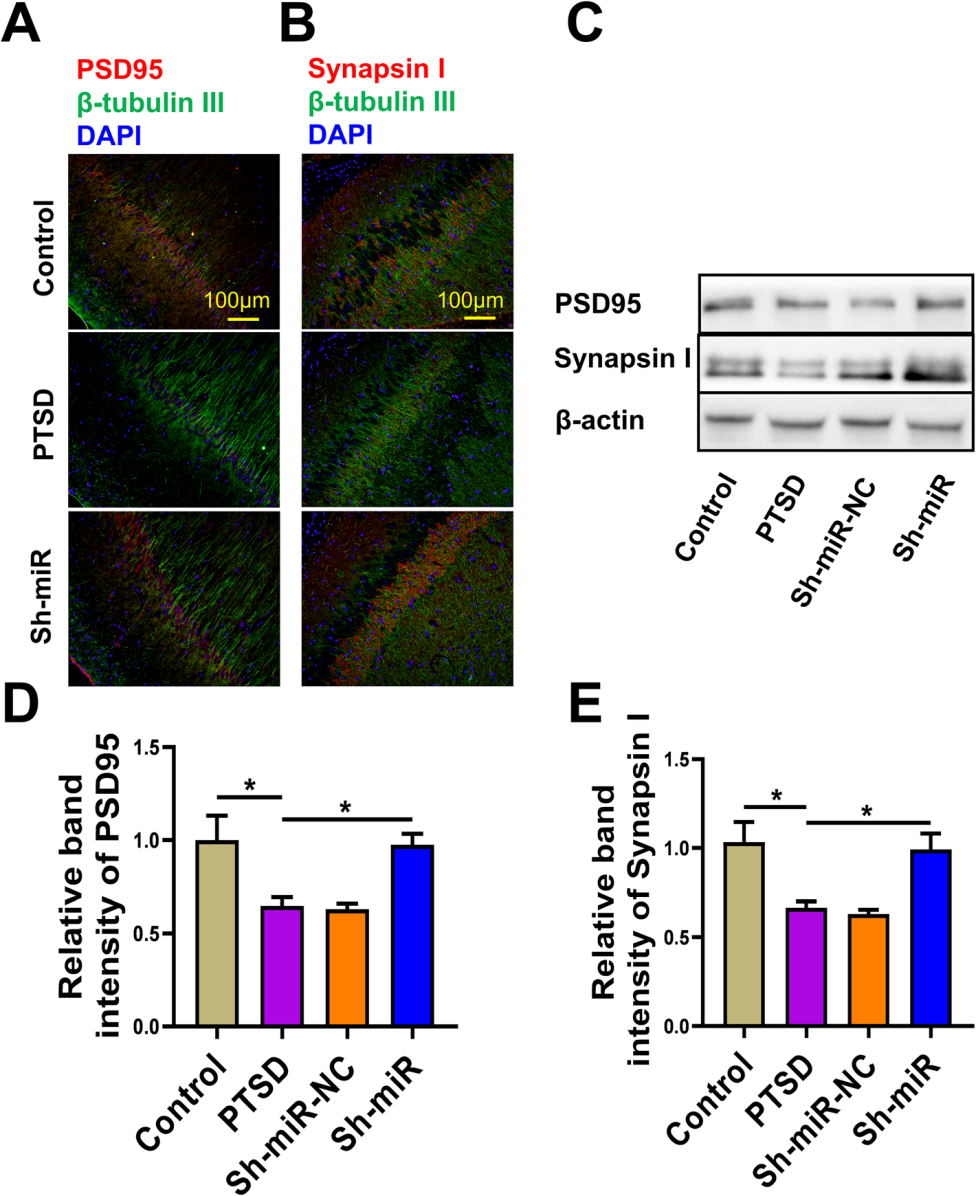
The effect of anti-miR-142 on the expression of synaptic proteins. Representative image indicating **a** PSD95 (red) and β-tubulin Ш (green), and **b** Synapsin I (red) and β-tubulin Ш (green) immunopositive cells in rat hippocampus of the control, PTSD, and Sh-miR groups. **c** The relative expression of PSD95 and Synapsin I by western blot analysis in rat hippocampus of the control, PTSD, Sh-miR-NC, and Sh-miR groups. Sample western blots for each protein are presented with β-actin as a loading control. The relative band intensity of **d** PSD95 and **e** Synapsin I protein in each group. *n* = 6 rats per genotype. Bar graphs represent the mean ± SEM of independent experimental triplicates. One-way ANOVA followed by Bonferroni’s post hoc test; *P < 0.05

PSD-95 is a scaffold protein of the enlarged head of dendritic spines, which forms functional synapses with presynaptic dendrites [30]. Synapsin I is involved in regulating the number of synaptic connections in response to extracellular signals. To investigate the effect of miR-142 inhibition on neuronal morphology, we evaluated the expression levels of PSD95 and synapsin I in the rat hippocampus by immunofluorescence staining and western blotting. Compared with the control group and the Sh-miR group, the percentage of PSD95 and synapsin I positive cells in β-tubulin III positive cells in the PTSD group was reduced, as shown in Fig. 2a and b. Lower PSD95 and synapsin I were found in the hippocampus of the PTSD group compared with the control group, but there was no significant difference between the Sh-miR and the control group (PSD95: *F*(3, 20) = 6.834, *P* = 0.0024, Fig. 2d; synapsin I: *F*(3, 20) = 7.915, *P* = 0.0011, Fig. 2e). These results indicated that miR-142 could regulate synaptic protein expression and might affect synaptic morphology and further affect neural function.

### MiR-142 silence rescued apoptosis of hippocampal neurons in PTSD rats

**Fig. 3:**
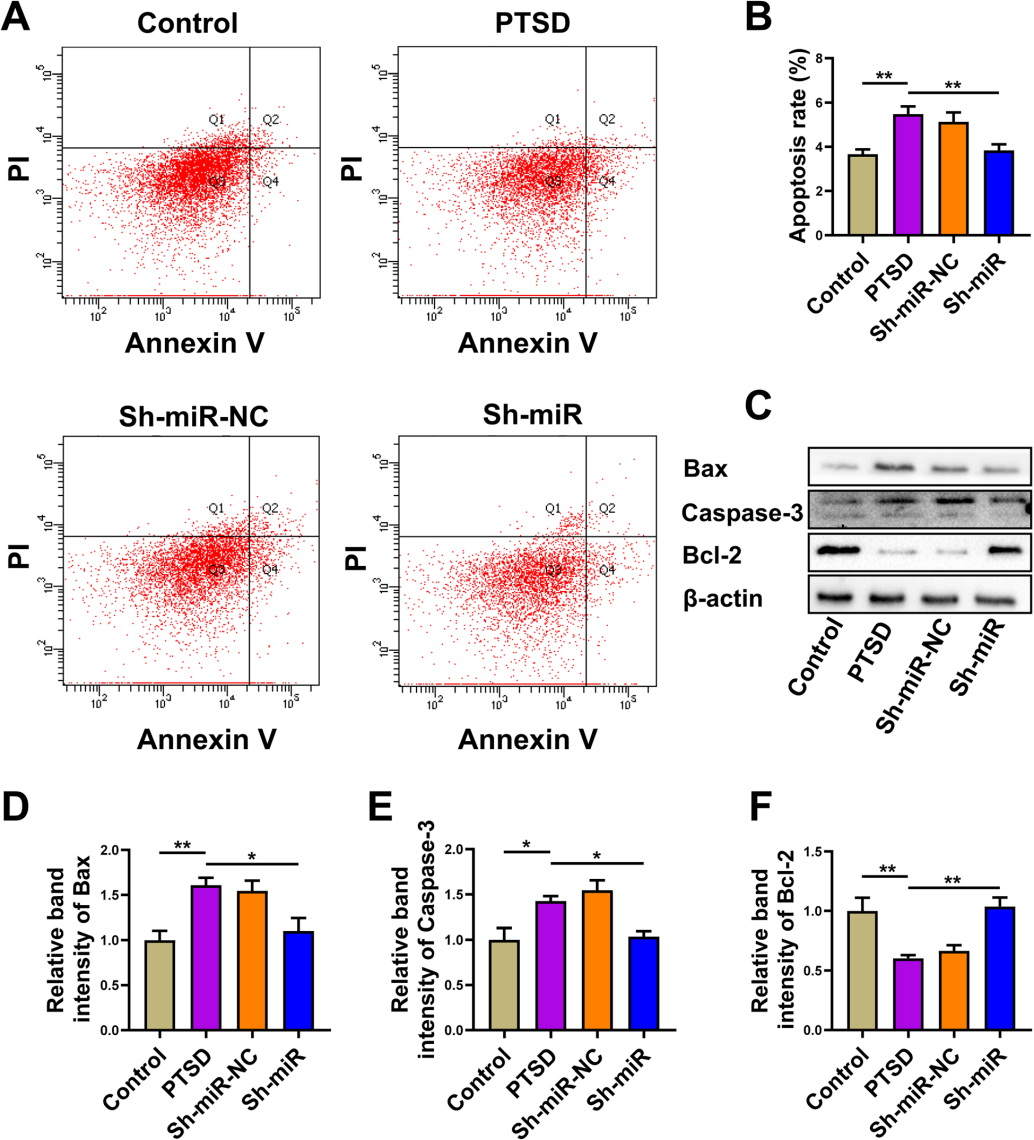
MiR-142 silence rescued the apoptosis of hippocampal neurons in PTSD rats. **a**, **b** The ratio of apoptosis in the control, PTSD, Sh-miR-NC, and Sh-miR groups after transfection with miR-142 inhibitor in rat hippocampus. **c** Western blot was used to detect the relative expression of Bax, caspase-3, and Bcl-2 in the hippocampus of rats in each group. Sample western blots for each protein are presented with β-actin as a loading control. The relative band intensity of **d** Bax, **e** caspase-3, and **f** Bcl-2 protein in each group. *n* = 6 rats per genotype. Bar graphs represent the mean ± SEM of independent experimental triplicates. One-way ANOVA followed by Bonferroni’s post hoc test; *P < 0.05, **P < 0.01

Next, we sought to determine the possible role of miR-142 in regulating neuronal apoptosis. As shown in Fig. 3a, we performed Annexin V-FITC/PI double-labeled flow cytometry to evaluate early apoptosis in the hippocampus by flow cytometry assay. The percentage of early apoptotic cells was increased in the hippocampus of SPS rats, while miR-142 silencing significantly the percentage of apoptotic cells in the Sh-miR group (*F*(3, 20) = 7.855, P = 0.0012, Fig. 3b). We further detected the levels of Bax, Caspase-3, and Bcl-2 in the hippocampus of rats in each group by western blotting.

The results showed that the expression of Bax and Caspase-3 was increased and the level of Bcl-2 was decreased in the hippocampus of rats exposed to SPS. The ratio of Bcl-2/Bax in the PTSD group decreased significantly. The administration of anti-miR-142 reduced the expression of Bax and Caspase-3 and upregulated the level of Bcl-2 (Bax: *F*(3, 20) = 7.525, *P* = 0.0015, Fig. 3d; Caspase-3: *F*(3, 20) = 8.578, *P* = 0.0007, Fig. 3e; Bcl-2: *F*(3, 20) = 9.526, *P* = 0.0004, Fig. 3f). The Bcl-2/Bax ratio increased significantly, which might be related to the anti-apoptotic effect of anti-miR-142.

The results of flow cytometry and western blotting showed that apoptosis in rat hippocampus exposed to SPS was significantly increased, while the level of apoptosis in stress rats treated with anti-miR-miR-142 decreased.

### Microglia were activated in the hippocampus of rats exposed to SPS procedure

We next sought to discover how anti-miR-142 regulated hippocampal neuromorphology and apoptosis in PTSD rats. Microglia can affect and regulate neuronal function by releasing soluble factors, including cytokines and prostaglandins [31]. Some studies also indicated that microglia regulated neuronal function through engulfment of synaptic and dendritic elements [32]. We aimed to discover whether anti-miR-142 could regulate the level of microglia and then affect the interaction between neurons and microglia.

**Fig. 4:**
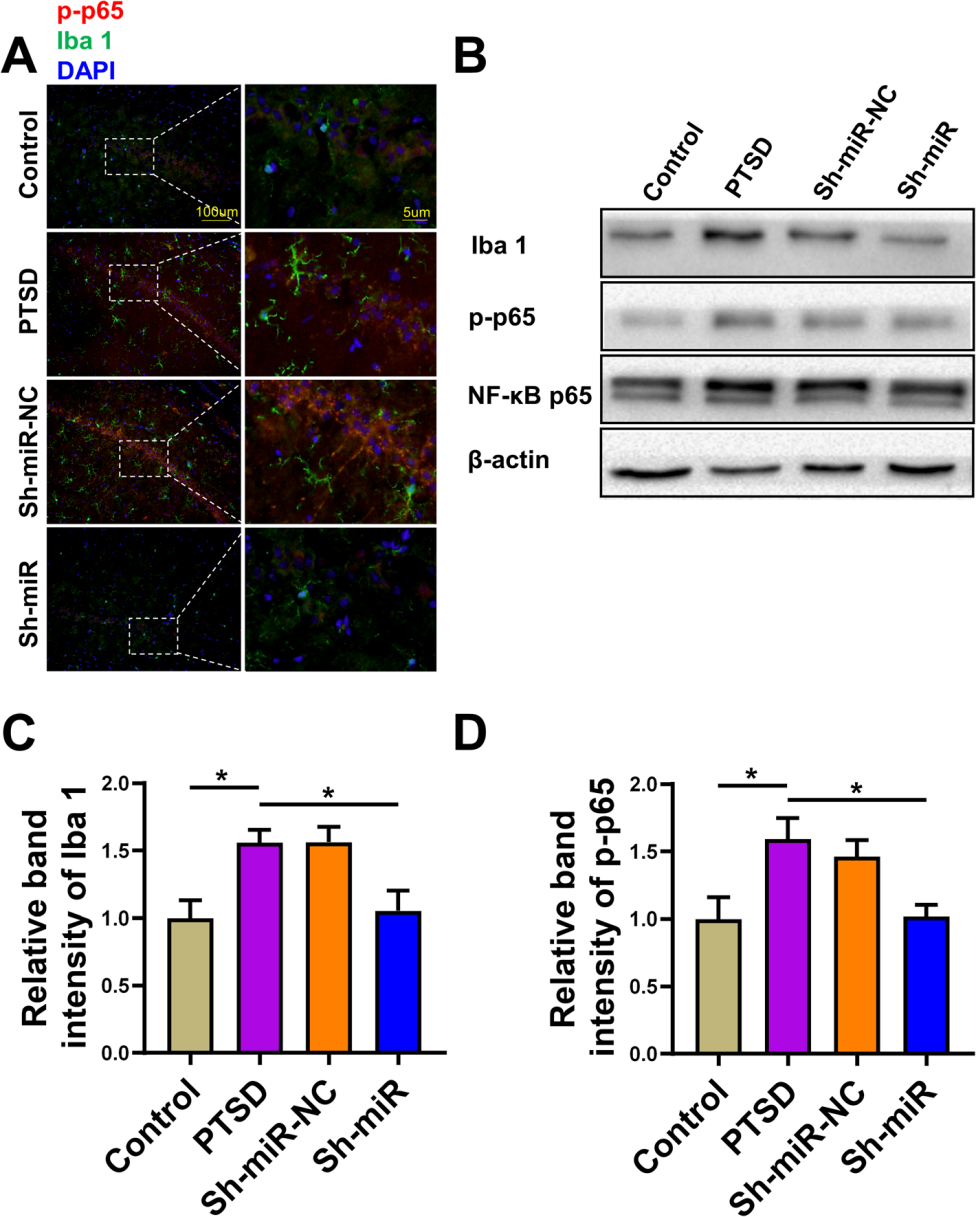
miR-142 could regulate the expression of Iba 1, p-p65 in rat hippocampus. **a** Representative image indicating Iba 1 (green) and p-p65 NF-κB (red) immunopositive cells in rat hippocampus of the control, PTSD, sh-miR-NC, and sh-miR groups. **b** The expression of Iba 1 and p-p65 NF-κB by western blot analysis in the hippocampi of rats in each group. Sample western blots for Iba 1 were presented with β-actin as a loading control, NF-κB p65 was used for a loading control to p-p65. The relative level of **c** Iba 1, and **d** p-p65 expression in each group. *n* = 6 rats per genotype. Bar graphs represent the mean ± SEM of independent experimental triplicates. One-way ANOVA followed by Bonferroni’s post hoc test; *P < 0.05

As shown in Fig. 4a, the proportion of Iba 1 positive cells in rat hippocampus exposed to the SPS procedure was significantly increased by immunofluorescence staining. Injection of anti-miR-142 significantly reduced Iba-1 levels in the hippocampus of PTSD rats (*F*(3, 20) = 6.213, *P* = 0.0037, Fig. 4c). These results suggested that SPS procedure might induce inflammation in the hippocampus of rats. This “neuroinflammation” caused by stress might reflect an inflammatory state called parainflammation, which seemed to be mainly mediated by tissue-resident macrophages, such as microglia [33]. NF-κB is a transcriptional factor that plays a crucial role in the expression of proinflammatory cytokines in microglia [34]. We next tested the phosphorylation level of the NF-κB subunit p65 (NF-κBp-p65), which resulted in its translocation into the nucleus and the transcription of various genes, including encoding multiple inflammatory cytokines like TNF-α and IL-1β [35]. The results showed that the phosphorylation level of p-p65 in the hippocampus of the PTSD group was increased, and the injection of anti-miR-142 significantly reversed this trend (*F*(3, 20) = 2.207, *P* = 0.0081, Fig. 4d).

These results indicated that SPS procedure might induce apoptosis and then activate microglia and NF-κB p65, which could be downregulated by miR-142 silencing.

### The effect of anti-miR-142 on the level of inflammatory cytokines in hippocampus of rats exposed to SPS procedure

**Fig. 5:**
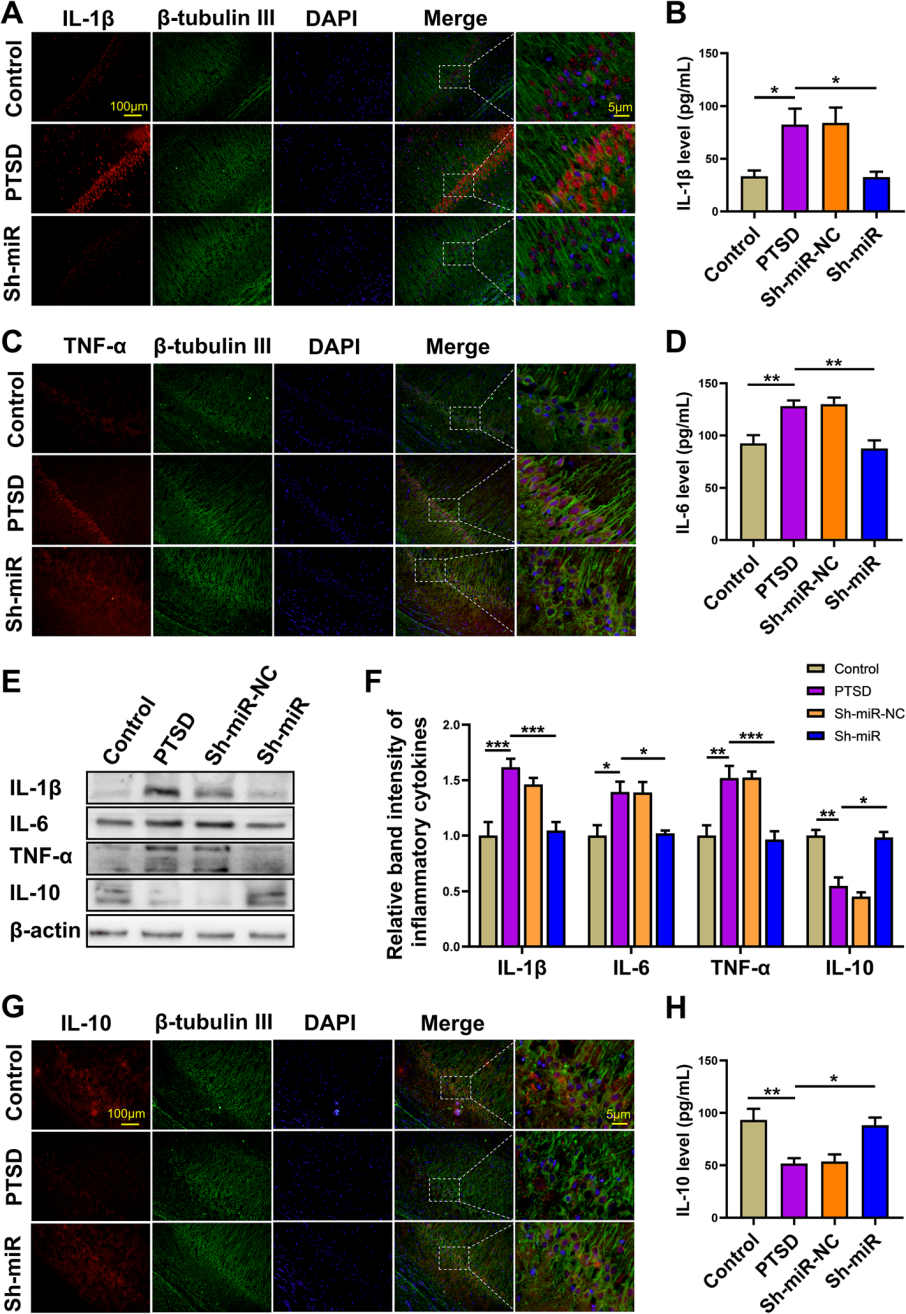
The effect of anti-miR-142 on pre-inflammatory chemokines and anti-inflammatory chemokines in PTSD rats. **a** Representative image indicating IL-1β (red) and β-tubulin III (green) positive cells in rat hippocampus of the control, PTSD, and Sh-miR groups. **b** IL-1β level in the control, PTSD, Sh-miR-NC, and Sh-miR groups tested by ELISA. **c** Representative image indicating TNF-α (red) and β-tubulin III (green) positive cells in rat hippocampus of the control, PTSD, and Sh-miR groups. **d** IL-6 level in rat hippocampus of the control, PTSD, Sh-miR-NC, and Sh-miR groups tested by ELISA. **e** The expression of IL-1β, IL-6, TNF-α, and IL-10 by western blot analysis in rat hippocampus of the control, PTSD, Sh-miR-NC, and Sh-miR groups. Sample western blots for all aimed protein were presented with β-actin as a loading control. **f** The relative level of inflammatory chemokines in each group. **g** Representative image indicating IL-10 (red) and β-tubulin III (green) positive cells in hippocampi of rats in control, PTSD, and Sh-miR groups. **h** IL-10 level in rat hippocampus of the control, PTSD, Sh-miR-NC, and Sh-miR groups tested by ELISA. *n* = 6 rats per genotype. Bar graphs represent the mean ± SEM of independent experimental triplicates. One-way ANOVA followed by Bonferroni’s post hoc test; *P < 0.05, **P < 0.01, ***P < 0.001

Within the brain, microglia are considered to be the principal source of proinflammatory cytokines and are pivotal regulators of neuroinflammation. They also contribute to homeostasis of the CNS where they induce or modulate a broad spectrum of cellular responses [36]. In order to investigate the effect of miR-142 inhibition on hippocampal inflammation in PTSD rats, the levels of the proinflammatory cytokines (IL-1β, TNF-α, and IL-6) and anti-inflammatory cytokine (IL-10) were measured by ELISA, western blot, or immunofluorescence. We found that the level of IL-1β, TNF-α, and IL-6 expression was significantly increased in the hippocampus of rats exposed to the SPS procedure compared to the levels in the control group (Fig. 5a–f). In contrast, IL-10 levels, which were considered anti-inflammatory and immunosuppressive factors, were significantly decreased (Fig. 5e–h). However, all the above trends could be significantly reversed by the administration of anti-miR-142.

These results suggested that SPS procedure could induce inflammatory responses and these trends could be reversed by miR-142 silence. These proinflammatory cytokines might influence neural morphology and function and then further affect animal behavior.

### Mir-142 silence upregulated the mature spines in cultured neurons

**Fig. 6:**
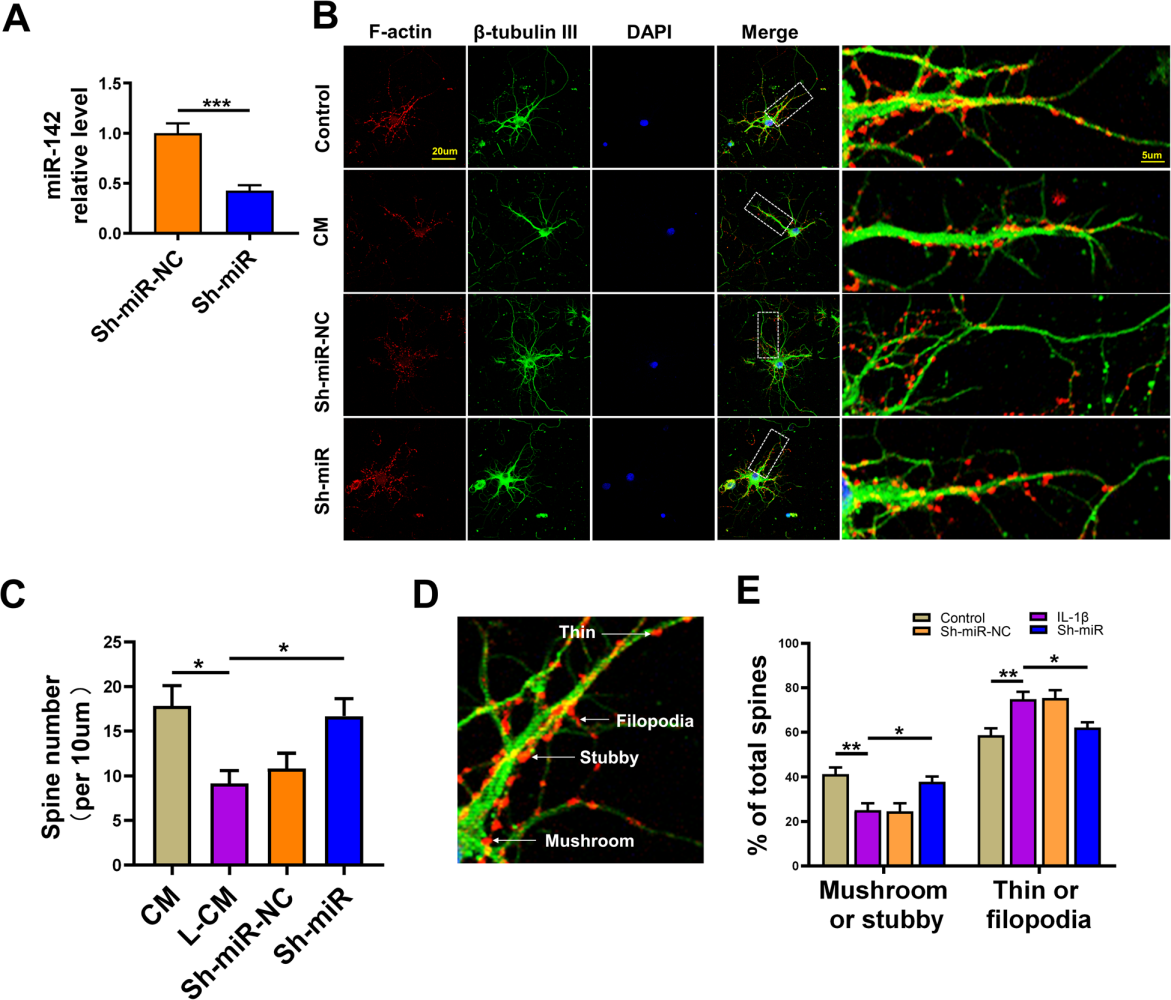
Anti-miR-142 enhanced dendritic spine development in rat hippocampal neurons exposed to the SPS procedure. **a** MiR-142 relative expression in primary hippocampal neurons 2 days after transfection of LV-anti-miR-142 by RT-qPCR. **b** Representative image indicating F-action and β-tubulin Ш immunopositive cells in the CM, L-CM, sh-miR-NC, and sh-miR groups. **c** Graphic representation of the number of dendritic spines in the CM, L-CM, sh-miR-NC, and sh-miR groups. **d** Four types (mushroom, stubby, thin, and filopodia) dendritic spines in hippocampal neurons. **e** Fraction of mushroom, stubby and thin, filopodia spines from primary hippocampal neurons in the CM, L-CM, sh-miR-NC, and sh-miR groups. One-way ANOVA followed by Bonferroni’s post hoc test; *P < 0.05, **P < 0.01, ***P < 0.001

Hippocampal neurons were cultured and exposed to LPS at a concentration of 100 ng/mL (L-CM) for 24 h and treated with microglia conditioned medium (CM) to examine the possible effect of miR-142 silencing on the morphology of neurons exposed to an inflammatory environment. Anti-miR-142 was successfully transfected in cells evaluated by RT-qPCR (Fig. 6a). Compared with the Sh-miR-NC group, the miR-142 level in the Sh-miR group was significantly decreased (*t* = 5.037, *P* = 0.0005, Fig. 6a). The immunofluorescence staining of F-actin and β-tubulin III was used to detect neuronal morphology. The results showed that the total number of spine in the L-CM group decreased, which could be reversed by anti-miR-142 (*F*(3, 20) = 5.180, *P* = 0.0082, Fig. 6d). Next, we calculated their ratio based on the shape of dendritic spines (Fig. 6e).

In the L-CM group, the number of mature spines [mushroom (detectable head > 0.6 μm in width) and stubby (length/width < 1)] was observed, and the proportion of immature spines [filopodia (length > 2 μm, no detectable head), thin (1 μm < length < 2 μm, detectable head < 0.6 μm in width)] was increased. These results indicated the effect of anti-miR-142 on dendritic spines and neuronal morphology.

### The effect of anti-miR-142 on FMRP expression and NF-κB p65 transportation

**Fig. 7:**
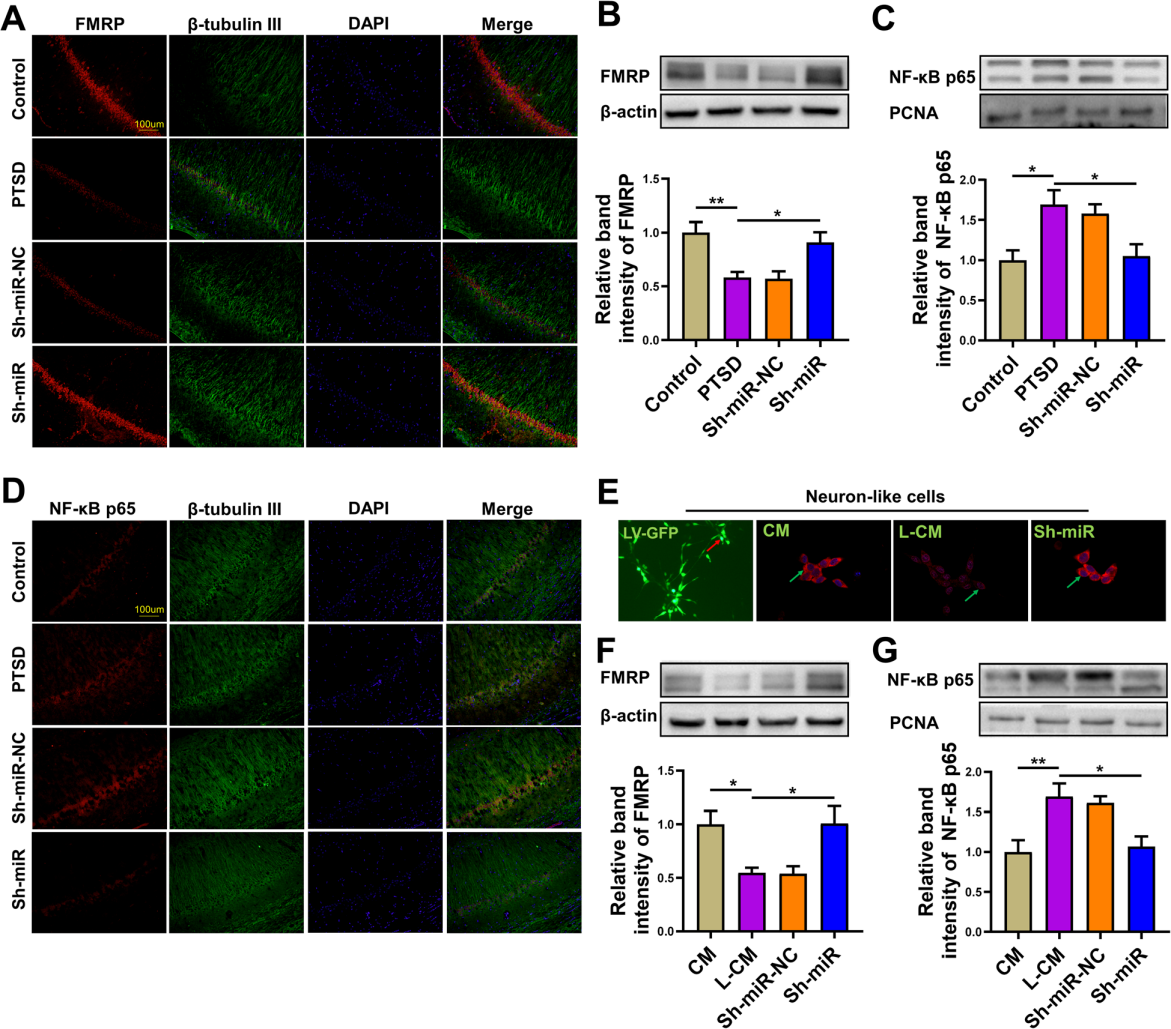
The effects of anti-miR-142 on the expression of FMRP, NF-κB p65 in rat hippocampus and neuron-like cells. **a** Representative image indicating FMRP and β-tubulin Ш immunopositive cells in rat hippocampus of the control, PTSD, sh-miR-NC, and sh-miR groups. **b** The relative level of FMRP in rat hippocampus of each group. Sample western blots for each protein are presented with β-actin as a loading control. **c** The relative level of NF-κB p65 expression in rat hippocampal nuclei of each group. Sample western blots for each protein are presented with PARN as a loading control for nuclear protein. **d** Representative image indicating NF-κB p65 and β-tubulin Ш immunopositive cells in rat hippocampus of each group. **e** The representative image of FMRP-positive neuron-like cells induced from PC12 cells in the CM, L-CM, and Sh-miR groups. The red arrow indicates GFP expression in neuron-like cells 2 days after transfection of LV-anti-miR-142 and the green arrows indicate PSD95 positive cells. **f** The relative level of FMRP in neuron-like cells in the CM, L-CM, Sh-miR-NC, and Sh-miR groups. Sample western blots for each protein are presented with β-actin as a loading control. **g** The relative level of NF-κB p65 in the nuclei of neuron-like cells of each group. Sample western blots for each protein are presented with PARN as a loading control for nuclear protein. *n* = 6 rats per genotype. Bar graphs represent the mean ± SEM of independent experimental triplicates. One-way or two-way ANOVA followed by Bonferroni’s post hoc test; *P < 0.05, **P < 0.01, ***P < 0.001

The mechanism by which anti-miR-142 affects hippocampal inflammation and neuronal morphology is unclear. FMRP, as an RNA-binding protein, is involved in regulating the local translation of several mRNAs that are critical for synapse elimination and maturation in the mammalian brain, and plays a crucial role in the development and function of neurons [13, 37, 38]. Interestingly, we predicted that miR-142-5p might have a binding site to the 3′-UTR region of FMRP through software analysis (http://www.targetscan.org/). Therefore, we further tested the level of FMRP in the hippocampus of rats by immunofluorescence staining and western blotting to explore the possible mechanism by which anti-miR-142 reduced PTSD-like behavior and downregulated the level of proinflammatory chemokines in PTSD rats. The results showed that compared with the PTSD group, the percentage of FMRP-positive neurons (both β-tubulin III and FMRP were stained) in the hippocampus of the Sh-miR group was increased (Fig. 7a).

Western blot analysis showed that the FMRP protein level in the hippocampus of the PTSD group was significantly downregulated, and anti-miR-142 could reverse this trend (*F*(3, 20) = 7.517, *P* = 0.0015, Fig. 7b). Nuclear factor kappa-B (NF-κB) is an inflammatory transcription factor, which could be activated by proinflammatory chemokines. After translocating into the nucleus, it upregulates the expression of proinflammatory immune response genes, including tumor necrosis factor (TNF)-α, interleukin (IL)-1β, and IL-6. It has been reported that NF-κB played an important role in synaptic plasticity and might be regulated by FMRP [39].

Next, we evaluated the expression level of p65, a subunit of NF-κB, in the hippocampal cell nucleus, and found that its expression was increased in the PTSD group, and anti-miR-142 could reverse this trend by inhibiting the transportation of p65 to the nucleus. As shown in Fig. 7d, the percentage of p65 positive cells (red) in the hippocampus of the PTSD group was higher than that of the control group, while it was significantly lower in the Sh-miR group. Western blot analysis was consistent with the immunofluorescence results of p65NF-κB, as shown in Fig. 7c (*F*(3, 20) = 6.167, *P* = 0.0038).

We also tested the levels of FMRP and NF-κB p65 in neuron-like cells induced by NGF-cultured PC12 cells exposed to L-CM to determine the role of miR-142 in regulating FMRP and NF-κB. The statistical results were consistent with those obtained in the tissue (FMRP: *F*(3, 20) = 5.795. *P* = 0.0051, Fig. 7f; p65: *F*(3, 20) = 7.217. *P* = 0.0018, Fig. 7g).

These results indicated that miR-142 might regulate NF-κB transportation by binding to the 3′-UTR region of FMRP, which may affect the morphology and function of rat hippocampal neurons.

### MiR-142 regulates NF-κB by binding with FMRP

**Fig. 8:**
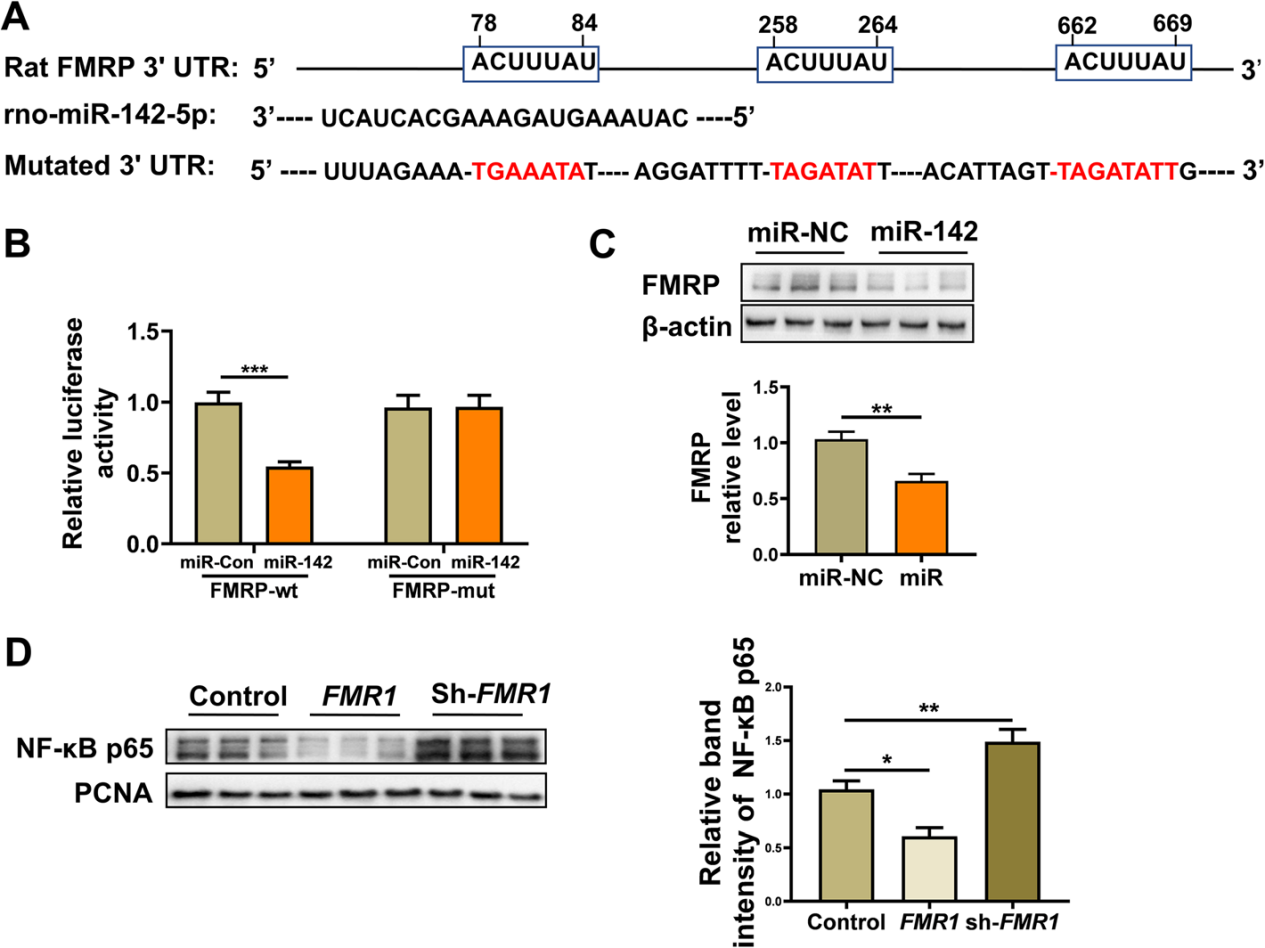
FMRP is a target gene of miR-142 verified in vitro. **a** Schematic illustration indicating the conserved seed match of FMRP 3′UTR to miR-142-5p. The putative binding sites were predicted by TargetScan. **b** Relative luciferase activity expressed with firefly/Renilla luciferase activity. Luciferase was fused to the 3′UTR of FMRP containing wild-type or a mutated seed sites in the presence of miR-control or miR-142-5p. **c** The expression of FMRP in neuron-like cells by western blot analysis and the relative band intensity of FMRP protein in the miR-NC and miR groups. **d** The expression of NF-κB p65 in the nuclei of neuron-like cells by western blot analysis and the relative band intensity of NF-κB p65 protein in the control, *FMR1,* and sh-*FMR1* groups. Bar graphs represent the mean ± SEM of independent experimental triplicates. *P < 0.05, **P < 0.01, ***P < 0.001

Studies have shown that microRNAs can inhibit gene expression by binding to the 3′-UTR site of target genes. To understand whether FMRP is one of the target genes of miR-142, we used bioinformatics analysis (http://www.targetscan.org/) and found that the 3′-UTR region of FMRP had three binding sites to miR-142-5P, which contained conserved seed sequences at positions 78-84, 258-264, and 662-669 (Fig. 8a).

Then, we further determined whether FMRP was a direct target of miR-142 by dual-luciferase reporter assay. We constructed the FMRP 3′-UTR of the microRNA-binding site downstream of the firefly luciferase gene. The results showed that in HEK 293 T cells co-transfected with wild-type vectors, relative luciferase activity was significantly reduced and miR-142 expression was enhanced (Fig. 8b; *P* < 0.001), while no reduction occurred when the miR-142 seed sequence was mutated.

To further verify that miR-142 regulates FMRP expression levels, LV-miR-142 or LV-miR-142 scramble were transfected into neuron-like cells and interested protein expression was tested by western blotting. The results showed that compared with the miR-NC group transfected with the LV-miR-142 scramble, the FMRP protein levels in the cells of the miR group transfected with LV-miR-142 were reduced (*t* = 4.130, *P* = 0.0020, Fig. 8c).

To investigate whether miR-142 modulates NF-κBp65 by binding to FMRP, we evaluated the expression level of NF-κB p65 in neuron-like nuclei transfected with *FMR1* or *sh-FMR1*. Compared with the control group, the expression level of NF-κBp65 in the FMR1 group was significantly reduced, while the expression level of NF-κBp65 in the sh-FMR1 group was significantly increased (*F*(2, 15) = 23.30. *P* < 0.0001, Fig. 8d).

These results suggested that FMRP was a direct target of miR-142, and miR-142 could inhibit the expression of FMRP by binding to its 3′-UTR site. miR-142 could regulate the expressive of NF-κB p65 in the nucleus by binding to FMRP.

## Discussion

In the present study, we found that miR-142 levels in rat hippocampus were higher than those in the control group 30 min and 7 days after exposure to the SPS procedure. Excessive miR-142 contributed to abnormal behavior in PTSD rats and downregulated the expression of synaptic proteins including PSD95 and synapsin I and might induce neural apoptosis in the hippocampus of animals. These neuronal abnormalities might be activated by microglia and induce the release of proinflammatory chemokines in the hippocampus. Administration of antisense miR-142-5p through the lateral ventricle could alleviate PTSD-like behaviors in rats exposed to the SPS paradigm, such as anxiety-like, depression-like, and abnormal memory.

This effect of anti-miR-142 might be related to the restoration of synaptic protein expression, neuronal apoptosis, inflammation, and further recovery of neural morphology and function. In addition, this effect might also be related to FMRP, an RNA-binding protein that could bind to miR-142, and the transportation of NF-κB was closely related to inflammation.

The microglial changes induced by the SPS procedure might directly promote the abnormal expression of synaptic proteins that was observed in the hippocampus of rats. Microglial cells could engulf synaptic elements and improve synaptic remodeling during early stages of neurodevelopment and responding to stress challenges such as PTSD [11, 40–42]. Indeed, some studies have shown that stress exposure triggers phagocytosis of synaptic microglia, thereby alleviating the disorder of anxiety and depression-related behavior in the chronic unpredictable stress model (CUS) and PTSD caused by shock exposure [40, 43]. The present study extended these results with another method of inducing stress, the SPS paradigm, in which animals exhibited significant PTSD-like behaviors such as anxiety, depression, and abnormal learning and memory abilities. The results indicated that microglia were activated, and the expression levels of synaptic proteins such as PSD95 and synapsin I were downregulated in the hippocampus by the SPS procedure, which was a brain region involved in regulating cognitive, emotional, and behavioral responses [44]. Soluble factors released by microglia, including cytokines and prostaglandins, can regulate neuronal responses under physiological and pathological conditions [31]. Our research showed that the SPS procedure could increase the release of proinflammatory chemokines, such as TNF-α, IL-1β, and IL-6, and reduce the levels of anti-inflammatory factors, such as IL-10. These proinflammatory chemokines released by microglia might affect neuronal function and neuroplasticity, as previously reported [45, 46].

The morphological plasticity of spines is closely related to the input of information such as learning and memory and functional plasticity including synaptic plasticity [47, 48]. Neuroinflammation may change the structure of synapses and the growth of dendritic spines, eventually leading to neuronal disease [49, 50]. Neuronal structural changes caused by changes in neuronal morphology and branching patterns have been reported to be closely related to neuronal diseases [51, 52].

In our study, we found that the SPS procedure could induce neuroinflammation and might lead to a decrease in the expression levels of synaptic proteins in hippocampal neurons, and further cause abnormal behavior in rats. We also detected a decrease in the ratio of the spine morphology of mushrooms and stubby in neurons after microglial conditioned medium treatment. Interestingly, the miR-142 silence could reverse the aforementioned trends in our research. These results indicated that miR-142 might regulate the interaction between hippocampal microglia and neurons and modulate the abnormal behavior of PTSD rats. However, the mechanism of this effect of miR-142 was not clear.

FMRP is an mRNA-binding protein with several typical selective binding sites for mRNA, and regulates translation efficiency and transportation of certain mRNAs [12, 53]. It is believed that FMRP regulates protein synthesis by providing a local source of newly synthesized protein in the synapse, and the absence of FMRP may lead to L-LTP deficiency in the frontal cortex and damage the traces of fear memory in *Fmr1* KO mice [14, 15]. Previous studies have shown that the loss of FMRP can lead to abnormal spine development, deficits in synaptic plasticity, hyperactivity, and learning and memory disorders [39]. In this study, we found that the SPS procedure downregulated the expression of FMRP in the hippocampus, which might be related to the upregulation of miR-142 that combined to FMRP. In PTSD rats, administration of anti-miR-142 through the lateral ventricle could alleviate PTSD-like behavior by upregulating FMRP. Meanwhile, the levels of synaptic proteins including PSD95 and synapsin I were also increased, and neuronal dysfunction might be restored. To check whether the effect of anti-miR-142 on neuronal morphology was related to FMRP, we transfected it and found that the proportion of mature spines in cultured neurons increased. The expression of FMRP in neuron-like cells induced by PC12 cells in the L-CM group decreased, and the culture medium was obtained from microglia exposed to 100 ng/mL LPS. This trend could also be reversed by miR-142 silence. These results indicated that miR-142 played a regulatory role in neural morphology and function, which may be related to FMRP.

Interestingly, we found that the transportation of NF-κB p65 into the nuclei of neurons was decreased by anti-miR-142 injection. Then, we further investigated whether there was a regulatory relationship between NF-κB and FMRP. We found that upregulation of FMR1 could inhibit the transportation of NF-κB into the nucleus of neuron-like cells. Abnormal expression of FMRP leads to abnormal development and impairment of dendritic spines due to activation of NF-κB transportation.

## Conclusion

In summary, we found that the interaction between neurons and microglia might be one of the mechanisms of behavioral abnormalities in PTSD rats, and miR-142 might be involved in the regulation of these two kinds of cells through the FMRP/NF-κB signaling pathway, which further indicated that miR-142 or FMRP might be a potential target for PTSD prevention or treatment. Further work should be undertaken to explore other detailed signaling cascades involved in the regulation of FMRP on neurons with the intervention of miR-142 silence. This work will provide evidence that miR-142 or FMRP may be a potential target for PTSD prevention or treatment.

## Data Availability

All data supporting the conclusions of this manuscript are provided in the text and figures.

## Abbreviations

PTSD: Post-traumatic stress disorder
SPS: Single-prolonged stress
NF-κB: Nuclear factor kappa-B
IL-1β: Interleukin-1β
IL-6: Interleukin-6
TNF-α: Tumor necrosis factor-α
CRP: C-reactive protein
ROS: Reactive oxygen species
RNS: Reactive nitrogen species
FMRP: Fragile X mental retardation protein
miRNAs: MicroRNAs
CNS: Central nervous system
CM: Conditioned medium
LPS: Lipopolysaccharide
FST: Forced swimming test
EPM: Elevated plus maze
OFT: Open field test
MWM: Morris water maze
HBSS: Hank’s balanced salt solution
L-CM: LPS-treated cells conditioned media
DMEM: Dulbecco’s modified Eagle medium
NGF: Nerve growth factor
PFA: Paraformaldehyde
RIPA: Radioimmunoprecipitation assay buffer
PVDF: Polyvinylidene fluoride
PE: Phycoerythrin
7-AAD: 7-Aminoactinomycin D
